# Hepatitis C virus and non-Hodgkin's lymphoma: findings from the Swiss HIV Cohort Study

**DOI:** 10.1038/sj.bjc.6603472

**Published:** 2006-11-14

**Authors:** S Franceschi, J Polesel, M Rickenbach, L Dal Maso, N M Probst-Hensch, C Fux, M Cavassini, B Hasse, A Kofler, B Ledergerber, P Erb, G M Clifford, and the Swiss HIV Cohort Study

**Affiliations:** 1 International Agency for Research on Cancer, 150 cours Albert Thomas, 69372 Lyon Cedex 08, France; 2 Aviano Cancer Center, Via Pedemontana occidentale 12, 33081 Aviano, Italy; 3 Coordination and Data Center, Swiss HIV Cohort Study, Mont-Paisible 16, CH-1011 Lausanne, Switzerland; 4 Molecular Epidemiology/Cancer Registry Zurich, University of Zurich, Sonneggstrasse 6, CH-8091 Zurich, Switzerland; 5 Division of Infectious Diseases, University Hospital Bern, Inselspital PKT2 B, CH-3010 Bern, Switzerland; 6 Division of Infectious Diseases, University Hospital Lausanne, Rue du Bugnon 46, CH-1011 Lausanne, Switzerland; 7 Division of Infectious Diseases and Hospital Epidemiology, University Hospital Zurich, Rämistrasse 100, CH-8091 Zurich, Switzerland; 8 Institute for Medical Microbiology, University of Basel, Petersplatz 10, CH-4051 Basel, Switzerland

**Keywords:** hepatitis C virus, non-Hodgkin's lymphoma, HIV, Switzerland

## Abstract

Infections with hepatitis C virus (HCV) and, possibly, hepatitis B virus (HBV) are associated with an increased risk of non-Hodgkin's lymphoma (NHL) in the general population, but little information is available on the relationship between hepatitis viruses and NHL among people with HIV (PHIV). We conducted a matched case–control study nested in the Swiss HIV Cohort Study (SHCS). Two hundred and ninety-eight NHL cases and 889 control subjects were matched by SHCS centre, gender, age group, CD4+ count at enrolment, and length of follow-up. Odds ratios (OR) and corresponding 95% confidence intervals (CI) were computed using logistic regression to evaluate the association between NHL and seropositivity for antibodies against HCV (anti-HCV) and hepatitis B core antigen (anti-HBc), and for hepatitis B surface antigen (HBsAg). Anti-HCV was not associated with increased NHL risk overall (OR=1.05; 95% CI: 0.63–1.75), or in different strata of CD4+ count, age or gender. Only among men having sex with men was an association with anti-HCV found (OR=2.37; 95% CI: 1.03–5.43). No relationships between NHL risk and anti-HBc or HBsAg emerged. Coinfection with HIV and HCV or HBV did not increase NHL risk compared to HIV alone in the SHCS.

People infected with HIV (PHIV) have an approximately 100-fold increased risk of non-Hodgkin's lymphoma (NHL), mainly high-grade B-cell NHL, compared to the general population (Dal Maso and Franceschi, 2003). The role of HIV seems to be indirect and related to the effect of the virus on immunoregulation. Conversely, the causal role of oncogenic viruses such as Epstein–Barr virus (EBV) and, in much rarer instances, Kaposi's sarcoma herpes virus in the onset of NHL in PHIV is well established (Jaffe *et al*, 2001).

An association between hepatitis C virus (HCV) infection and NHL in the general population has emerged in the last decade (Gasparotto *et al*, 2002; Negri *et al*, 2004) and a meta-analysis of 17 studies from eight countries has shown a pooled relative risk of 2.5 (95% confidence interval (CI): 2.1–3.0) (Dal Maso and Franceschi, in press).

Owing to shared routes of transmission, HCV and HIV coinfection is common and is associated with higher HCV RNA levels and increased risk of progression of HCV-related liver disease than in the presence of HCV infection alone (Sulkowski *et al*, 2000). Some investigators also attempted to evaluate whether coinfection with HCV increased the risk of NHL among PHIV, but did not find any association (Besson *et al*, 1999; Levine *et al*, 1999; Engels *et al*, 2002; de Sanjosé *et al*, 2004; Waters *et al*, 2005). None of these studies, however, included more than 12 cases of NHL positive for both HIV and HCV (Besson *et al*, 1999; Levine *et al*, 1999; de Sanjosé *et al*, 2004; Waters *et al*, 2005).

We have therefore, carried out a much larger case–control study nested in the Swiss HIV Cohort Study (SHCS) to assess the relationship between HCV infection and NHL in PHIV. On account of some reports of increased NHL risk in HIV-negative individuals seropositive for hepatitis B surface antigen (HBsAg) (Kim *et al*, 2002; Talamini *et al*, 2004), we also evaluated markers of HBV infection.

## MATERIALS AND METHODS

### Subjects

The SHCS is an ongoing study that has been enrolling PHIV since 1988, with some retrospective enrolment going back to 1984, from seven large hospitals in Switzerland (www.shcs.ch). Follow-up visits take place every 6 months and clinical events, such as opportunistic diseases, selected cancers (i.e. Kaposi's sarcoma, NHL, cervical cancer and Hodgkin's lymphoma), and death have always been recorded.

The database used for the present study included information recorded for the SHCS up to October 2004, and has been supplemented with independent information on cancer occurrence from a record-linkage study between the SHCS and eight Swiss Cantonal Cancer Registries (Clifford *et al*, 2005). Cases included in the present nested case–control study were PHIV who developed NHL, and for whom results on antibodies against HCV (anti-HCV), or, in their absence, a blood sample taken before NHL diagnosis (serum or plasma) to test for anti-HCV, was available. Out of a total of 492 NHL cases identified, 77 were excluded because information on some matching criteria (see below) was not available, and 117 because HCV serology status was impossible to obtain. The present study finally included 298 NHL cases (Table 1). Primary brain lymphomas (PBL) were distinguished from non-PBLs, and non-PBLs were classified, when possible, by major histological subtype (i.e. diffuse large B-cell lymphomas, Burkitt lymphomas or other) using the World Health Organisation classification of lymphoid neoplasms (Jaffe *et al*, 2001).

For each NHL case three control subjects were chosen at random among SHCS participants who had at least the same length of follow-up as the matched-case, and for whom HCV serology, or a blood sample to perform the test, was available. Matching criteria were: (1) centre of enrolment in the SHCS; (2) gender; (3) age group (in 5-year groups from 16 to 20 to ⩾65 years); and (4) CD4+ count (<100, 100–199, 200–499, ⩾500 cells *μ*l^−1^) at enrolment. For five control subjects, the retrieved blood sample was inadequate for anti-HCV testing, leaving 889 control subjects available for the present study (Table 1). Follow-up was censored at the reference date (i.e. the date of NHL diagnosis for cases, and the date occurring after a similar length of follow-up for matched control subjects).

As incidence of NHL was much greater in the early part of the SHCS and we did not match for year of enrolment, median year of enrolment was earlier for NHL cases (1991; interquartile range: 1990–1994) than for control subjects (1995; 1991–1998). Median year at cancer diagnosis for NHL cases was 1995 (interquartile range: 1993–1998).

HIV-transmission category was classified into three groups: (1) intravenous drug users (IDUs), (2) men having sex with men (MSM), and (3) heterosexual and others. ‘Others’ (i.e. transmission through blood and blood products and unknown transmission category) included only 14 NHL cases and 36 control subjects.

This study was approved by the local ethical committees of the clinics collaborating with the SHCS and of the International Agency for Research on Cancer. Written informed consent was obtained from all SHCS participants.

## SEROLOGY

All hepatitis virus markers were measured in blood samples taken before NHL diagnosis or the corresponding reference date for controls. As systematic anti-HCV testing in the SHCS started in April 1998, anti-HCV results could not be obtained from medical records for 172 (57.7%) NHL cases and 261 (29.4%) control subjects. New HCV testing on stored serum aliquots was, therefore, performed at the Institute for Medical Microbiology, University of Basel, Switzerland using third–generation ELISA (AxSYM anti-HCV, Abbott Diagnostic Division, Weisbaden, Germany). Reactive results were confirmed by immunoblot. Among 51 PHIV for whom anti-HCV results were available from both medical records and new HCV testing, anti-HCV status agreed in 50.

Information on antibodies against hepatitis B core antigen (anti-HBc) came from SHCS records for 100 NHL cases and 539 control subjects. New testing was performed on stored serum aliquots from 197 NHL cases and 347 control subjects, using microparticle enzyme immunoassay (AxSYM core TM, Abbott Diagnostic Division). Anti-HBc status could not be evaluated in one NHL case and three controls whose anti-HCV status was known.

For HBsAg, information came from SHCS records for 82 NHL cases and 472 control subjects. New testing was performed on stored serum aliquots from 210 NHL cases and 371 control subjects, using microparticle immunoenzyme assay (AxSYM HBsAg version 2.0, Abbott Diagnostic Division). Hepatitis B surface antigen status could not be evaluated in six NHL cases and 46 controls whose anti-HCV status was known.

Testing for antibodies against HBsAg and against HBe antigen was not systematically carried out in the SHCS, and testing for HCV RNA started only in February 2002. Thus, these three additional viral markers were not considered in the present study. The CD4+ count was measured by flow cytometry.

### Statistical analysis

Conditional logistic regression was used to calculate odds ratios (ORs) and corresponding 95% CIs. All analyses were conditioned on all matching variables (see Subjects) and adjusted for HIV-transmission category and year of enrolment (before 1990, 1990–1995, after 1995). Additional adjustment for highly active antiretroviral therapy (HAART) use did not substantially change the ORs reported. Unconditional logistic regression was used for the analyses stratified by HIV transmission category, adjusted for all matching variables and year of enrolment.

Heterogeneity of the ORs between strata of selected variables was tested by comparing the overall maximum-likelihood estimate for anti-HCV seropositivity to stratum-specific maximum-likelihood estimates. The test statistic was compared to the *χ*^2^ distribution with degrees of freedom equal to the number of strata minus one (Rothman and Greenland, 1998).

### RESULTS

Table 1 shows the distribution of NHL cases and control subjects by matching variables. Among the seven SHCS centres, Zurich alone contributed 44% of study participants. The majority of study participants were male (83%) and 48% were between 30 and 44 years of age. Forty per cent had a CD4+ count at enrolment below 200 cells *μ*l^−1^, and in 31% follow-up before the reference date was 60 months or longer (Table 1).

**Figure 1:**
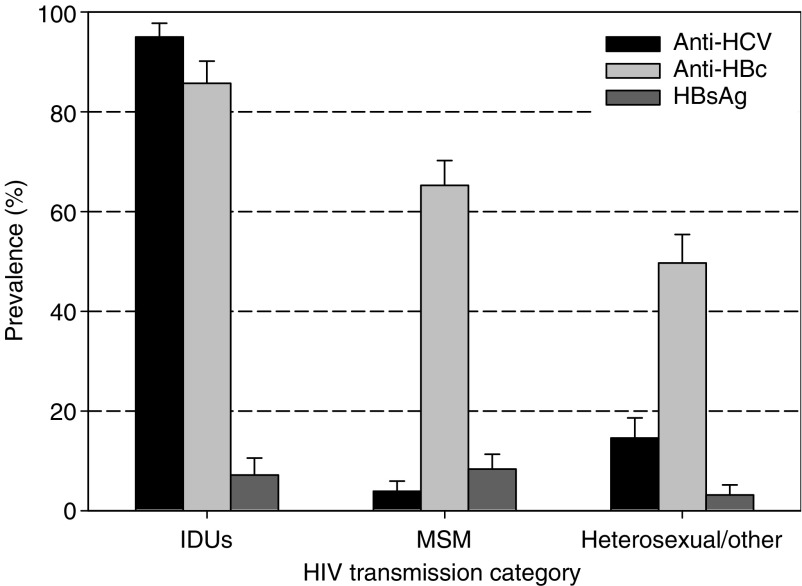
Seropositivity for antibodies against hepatitis C virus (anti-HCV), anti-HBc and hepatitis B surface antigen (HbsAg) in control subjects by HIV-transmission category. Swiss HIV Cohort Study, 1984–2004. IDU: intravenous drug users; MSM: men having sex with men.

Figure 1 shows seropositivity for the three hepatitis virus markers by HIV transmission category among control subjects. Anti-HCV was detected in 95.0% of IDUs, 3.9% of MSM and 14.6% of heterosexuals and other. Seropositivity for anti-HBc was nearly as frequent as seropositivity for anti-HCV among IDUs, but substantially higher than anti-HCV among MSM (65.3%) and heterosexuals and other (49.6%). Seropositivity for HBsAg was found in 7.2% of IDUs, 8.4% of MSM and 3.2% of heterosexuals and other.

None of the three hepatitis virus markers considered showed an association with NHL risk (Table 2). ORs were 1.05 (95% CI: 0.63–1.75) for anti-HCV, 0.85 (95% CI: 0.61–1.18) for anti-HBc and 0.62 (95% CI: 0.32–1.20) for HBsAg seropositivity.

Table 3 shows the influence of anti-HCV seropositivity on NHL risk in separate strata of CD4+ count at enrolment, age, gender and HIV transmission category. Anti-HCV^+^ PHIV did not show an increased NHL risk compared to anti-HCV^−^ PHIV in any separate stratum except for HIV transmission category, where a significant association between anti-HCV and NHL risk emerged among MSM (OR=2.37; 95% CI: 1.03–5.43). In no instance, however, was the effect of anti-HCV seropositivity on NHL risk significantly heterogeneous across the strata of the variables considered (Table 3).

Seropositivity for anti-HBc and HBsAg was not associated with NHL risk in any stratum of CD4+ count at enrolment, age, gender or HIV-transmission category (data not shown).

**Figure 2:**
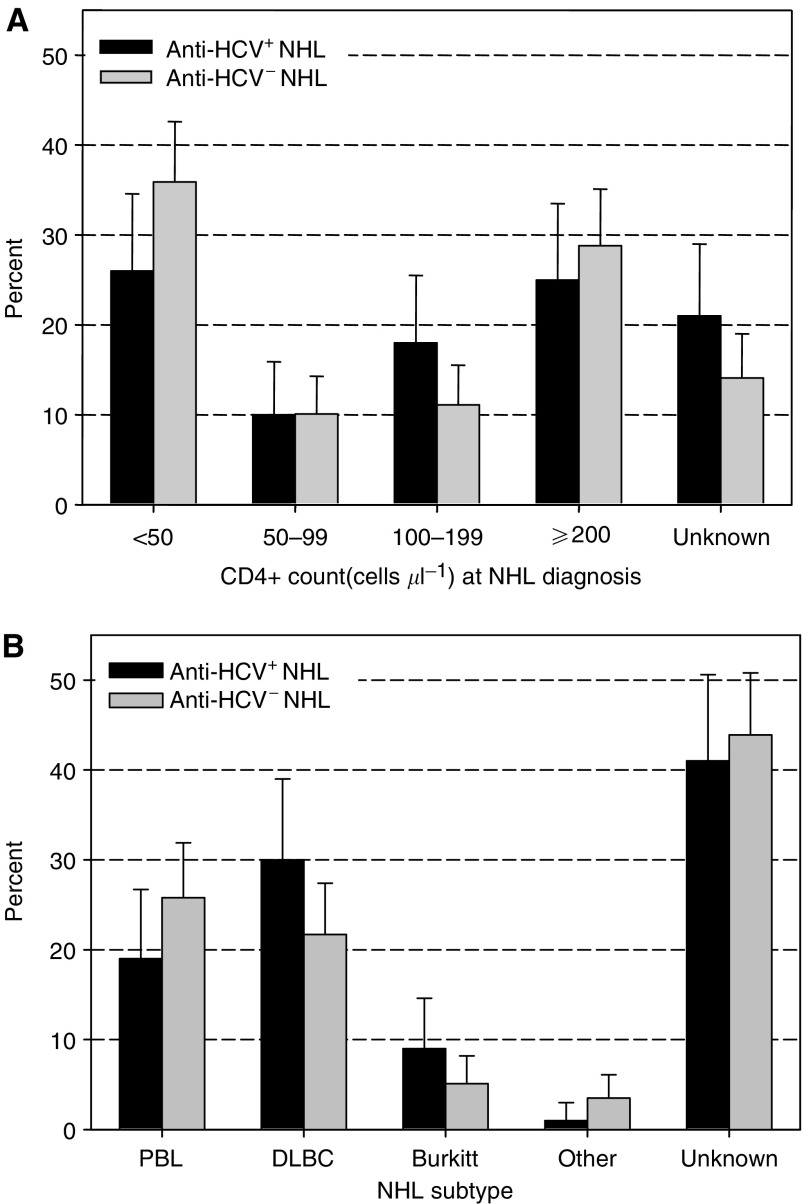
Comparison of percent distribution of CD4+ counts at NHL diagnosis (**A**) and NHL subtype (**B**) between 100 anti-HCV^+^ and 198 anti-HCV^−^ NHL cases. Swiss HIV Cohort Study, 1984–2004. Anti-HCV: antibodies against hepatitis C virus; PBL: Primary brain lymphoma; DLBC: diffuse large B-cell NHL. ‘Unknown’ refers to no CD4+ count result within 6 months before cancer diagnosis.

Figure 2 shows the percent distribution of CD4+ count at lymphoma diagnosis and NHL subtype separately among anti-HCV^+^ and anti-HCV^−^ NHL cases. Although 95% CIs always overlapped, a slightly lower proportion of anti-HCV^+^ than anti-HCV^−^ NHL cases had less than 50 CD4+ cells *μ*l^−1^ at cancer diagnosis (26.0 *vs* 35.9%, respectively), or were diagnosed with PBL (19.0 *vs* 25.8%, respectively).

## DISCUSSION

Seropositivity for HBV and HCV did not increase NHL risk among PHIV in the SHCS. Our findings on HCV, the most studied hepatitis virus in respect to NHL risk (Dal Maso and Franceschi, in press), are consistent with previous studies of PHIV that also did not show an association (Besson *et al*, 1999; Levine *et al*, 1999; Waters *et al*, 2005). Compared to earlier reports on the topic, our study was, however, much larger, allowing stratification by, and more accurate allowance for, other correlates of NHL risk.

As expected, levels of seropositivity for hepatitis viruses were high among PHIV, most notably among IDUs. The higher anti-HBc/anti-HCV seropositivity ratio among MSM (16.7) and heterosexuals and other (3.4) compared to IDUs (0.90) confirms the much greater efficiency of the sexual-route of transmission for HBV than HCV (IARC, 1994).

We were not able to review the slides of NHL cases, and histological type was not specified in 43% of the cases, thus limiting the chance of detecting qualitative differences between anti-HCV^+^ and anti-HCV^−^ lymphomas. We did detect, however, a slight under-representation of anti-HCV^+^ compared to anti-HCV^−^ cases among NHL occurring at CD4+ counts below 50 cells *μ*l^−1^ and among PBL, which are the two lymphoma subtypes where EBV is known to be most strongly implicated (Jaffe *et al*, 2001). Among transplant patients, for instance, the majority of NHL is associated with EBV, but NHL has also been reported in EBV^−^ patients and tends to occur longer after organ transplant, when immunosuppression is milder, than in EBV^+^ patients (Leblond *et al*, 2001).

As among transplant patients, the strong role of immunodeficiency and EBV in NHL (Jaffe *et al*, 2001) makes the evaluation of possible weak risk factors such as HCV infection much more difficult among PHIV than in the general population (Negri *et al*, 2004). A hint of an association between NHL risk and HCV infection emerged among MSM, who had lower levels of anti-HCV seropositivity than heterosexuals and IDUs. Indeed, nearly all IDUs were anti-HCV^+^, thus preventing any meaningful evaluation of the effect of HCV infection on NHL risk. Furthermore, MSM (median age 38) were older than subjects in the other HIV-transmission categories (median age 31 and 35 for IDUs and heterosexuals and other, respectively). A two-fold increased NHL risk was also found among anti-HCV^+^ PHIV aged 45 years or older, although it did not reach statistical significance. Older age may be a correlate of longer exposure to HCV, and, hence, higher risk of HCV-related complications including NHL. Indeed, the relative risk for cirrhosis in anti-HCV^+^ *vs* anti-HCV^−^ PHIV increased between the pre-HAART and HAART era (Giordano *et al*, 2004) and the excess of hepatocellular carcinoma currently seen in PHIV (Clifford *et al*, 2005) had not clearly emerged earlier in the epidemic (Beral and Newton, 1998).

An important strength of the SHCS is the fact that it is very representative of PHIV in Switzerland. It has been estimated that, since the beginning of the HIV epidemic, 48% of PHIV, and 68% of people diagnosed with AIDS in Switzerland have been enrolled in the SHCS (www.shcs.ch). Weaknesses of the SHCS include incomplete information on time of HIV seroconversion, which prevented us from using years of follow-up as an exact proxy for duration of HIV infection. In addition, no information was available on EBV infection and on the activity of HCV infection, that is the presence of serum HCV RNA. The vast majority of persons acutely infected with HCV develop chronic persistent viraemia and, especially among PHIV, anti-HCV seropositivity is likely to correspond to chronic HCV infection (Sulkowski *et al*, 2000).

In conclusion, coinfection with HIV and HCV or HBV did not increase NHL risk compared to HIV alone in the SHCS. However, as the life expectancy of PHIV increases, the influence of HCV infection on cancer risk deserves further study as the high prevalence of HCV could result in huge attributable risks, even in the presence of weak relative risks.

## Figures and Tables

**Table 1:** Distribution of 298 NHL cases and 889 control subjects according to matching variables (Swiss HIV Cohort Study, 1984–2004)

	**NHL cases**		**Control subjects**	
	******N******	**(%)**	******N******	**(%)**
*Centre*				
Basel	28	(9.4)	84	(9.5)
Bern	23	(7.7)	68	(7.7)
Geneva	42	(14.1)	125	(14.1)
St Gallen	9	(3.0)	27	(3.0)
Ticino	8	(2.7)	24	(2.7)
Vaud	57	(19.1)	169	(19.0)
Zurich	131	(44.0)	392	(44.1)
				
*Gender*				
Male	246	(82.6)	733	(82.5)
Female	52	(17.5)	156	(17.6)
				
*Age at enrolment (years)*				
<30	83	(27.9)	249	(28.0)
30–44	141	(47.3)	425	(47.8)
⩾45	74	(24.8)	215	(24.2)
				
*CD4*+ *at enrolment (cells μl*^−*1*^)				
<100	71	(23.8)	209	(23.5)
100–199	47	(15.8)	142	(16.0)
200–499	120	(40.3)	357	(40.2)
⩾500	60	(20.1)	181	(20.4)
				
*Duration of follow-up (months)*				
<24	100	(33.6)	297	(33.4)
24–59	107	(35.9)	320	(36.0)
⩾60	91	(30.5)	272	(30.6)

NHL=non-Hodgkin's lymphoma.

**Table 2:** ORs and corresponding 95% CIs for NHL by presence of hepatitis virus markers (Swiss HIV Cohort Study, 1984–2004)

	**NHL cases**		**Control subjects**		
**Seropositivity**	******N******	**(%)**	******N******	**(%)**	**OR (95% CI)^a^**
*Anti-HCV*					
No	198	(66.4)	605	(68.1)	1^b^
Yes	100	(33.6)	284	(32.0)	1.05 (0.63–1.75)
					
*Anti-HBc*					
No	101	(34.0)	305	(34.4)	1^b^
Yes	196	(66.0)	581	(65.6)	0.85 (0.61–1.18)
					
*HBsAg*					
No	279	(95.6)	790	(93.7)	1^b^
Yes	13	(4.5)	53	(6.3)	0.62 (0.32–1.20)

Anti-HCV=antibodies against hepatitis C virus; anti-HBc=antibodies against hepatitis B core antigen; HBsAg=antibodies against hepatitis B surface antigen; NHL=non-Hodgkin's lymphoma; OR=odds ratios; CI=confidence intervals.

a Estimated from conditional logistic regression analysis, conditioned on centre, gender, age group, and CD4+ count at enrolment and adjusted for HIV-transmission category and year of enrolment.

b Reference category.

**Table 3:** ORs and corresponding 95% CIs for NHL by presence of anti-HCV in strata of selected matching variables and HIV-transmission category (Swiss HIV Cohort Study, 1984–2004)

	**NHL cases**		**Control subjects**		
	***N***	**(%)**	***N***	**(%)**	**OR (95% CI)^a^**
*CD4*+ *at enrolment (cells μl*^−*1*^)					
<100					
Anti-HCV^−^	54	(76.1)	152	(72.7)	1^b^
Anti-HCV^+^	17	(23.9)	57	(27.3)	1.47 (0.47–4.53)
					
100–199					
Anti-HCV^−^	23	(48.9)	96	(67.6)	1^b^
Anti-HCV^+^	24	(51.1)	46	(32.4)	2.04 (0.67–6.20)
					
200–499					
Anti-HCV^−^	83	(69.2)	240	(67.2)	1^b^
Anti-HCV^+^	37	(30.8)	117	(32.8)	0.64 (0.26–1.60)
					
⩾500					
Anti-HCV^−^	38	(63.3)	117	(64.6)	1^b^
Anti-HCV^+^	22	(36.7)	64	(35.4)	1.06 (0.36–3.15)
Test for heterogeneity					*χ*_3_^2^=1.49; *P*=0.68
					
*Age group (years)*					
<30					
Anti-HCV^−^	50	(60.2)	136	(54.6)	1^b^
Anti-HCV^+^	33	(39.8)	113	(45.4)	0.62 (0.24–1.62)
30–44					
Anti-HCV^−^	82	(58.2)	267	(62.8)	1^b^
Anti-HCV^+^	59	(41.8)	158	(37.2)	1.04 (0.49–2.21)
					
⩾45					
Anti-HCV^−^	66	(89.2)	202	(94.0)	1^b^
Anti-HCV^+^	8	(10.8)	13	(6.1)	2.21 (0.73–6.70)
Test for heterogeneity					*χ*_2_^2^=1.56; *P*=0.46
					
*Gender*					
Female					
Anti-HCV^−^	26	(50.0)	88	(56.4)	1^b^
Anti-HCV^+^	26	(50.0)	68	(43.6)	1.07 (0.34–3.31)
					
Male					
Anti-HCV^−^	172	(69.9)	517	(70.5)	1^b^
Anti-HCV^+^	74	(30.1)	216	(29.5)	1.05 (0.59–1.87)
Test for heterogeneity					*χ*_1_^2^=0.00; *P*=0.98
					
*HIV-transmission category*					
Intravenous drug users					
Anti-HCV^−^	6	(7.5)	12	(5.0)	1^b^
Anti-HCV^+^	74	(92.5)	227	(95.0)	0.37 (0.11–1.22)^c^
					
Men having sex with men					
Anti-HCV^−^	132	(90.4)	341	(96.1)	1^b^
Anti-HCV^+^	14	(9.6)	14	(3.9)	2.37 (1.03–5.43)^c^
					
Heterosexuals and others					
Anti-HCV^−^	60	(83.3)	252	(85.4)	1^b^
Anti-HCV^+^	12	(16.7)	43	(14.6)	1.02 (0.44–2.36)^c^
Test for heterogeneity					*χ*_2_^2^=3.34; *P*=0.19

anti-HCV=antibodies against hepatitis C virus; HIV=human immunodeficiency virus; NHL=non-Hodgkin's lymphoma; OR=odds ratios; CI=confidence intervals.

a Estimated from conditional logistic regression analysis, conditioned on centre, gender, age group, and CD4+ count at enrolment and adjusted for HIV-transmission category and year of enrolment;

b Reference category.

c Estimated from unconditional logistic regression analysis, adjusted as in footnote a.
